# A Transcriptomic Network Identified in Uninfected Macrophages Responding to Inflammation Controls Intracellular Pathogen Survival

**DOI:** 10.1016/j.chom.2013.08.004

**Published:** 2013-09-11

**Authors:** Lynette Beattie, Micely d’El-Rei Hermida, John W.J. Moore, Asher Maroof, Najmeeyah Brown, Dimitris Lagos, Paul M. Kaye

**Affiliations:** 1 Centre for Immunology and Infection, Hull York Medical School and Department of Biology, University of York, York YO10 5DD, UK

## Abstract

Intracellular pathogens modulate host cell function to promote their survival. However, in vitro infection studies do not account for the impact of host-derived inflammatory signals. Examining the response of liver-resident macrophages (Kupffer cells) in mice infected with the parasite *Leishmania donovani*, we identified a transcriptomic network operating in uninfected Kupffer cells exposed to inflammation but absent from Kupffer cells from the same animal that contained intracellular *Leishmania*. To test the hypothesis that regulated expression of genes within this transcriptomic network might impact parasite survival, we pharmacologically perturbed the activity of retinoid X receptor alpha (RXRα), a key hub within this network, and showed that this intervention enhanced the innate resistance of Kupffer cells to *Leishmania* infection. Our results illustrate a broadly applicable strategy for understanding the host response to infection in vivo and identify *Rxra* as the hub of a gene network controlling antileishmanial resistance.

## Highlights

• *Leishmania* infection rapidly activates infected and uninfected Kupffer cells in mice
• Transcriptomics of inflamed and infected KC uncover distinct and overlapping networks
• A network centered on RXRα is uniquely activated in inflammation-exposed uninfected KCs
• Manipulation of RXRα function leads to a reduction in early parasite burden

## Introduction

Intracellular pathogens have evolved over many millions of years to coexist in the face of a wide array of host defense mechanisms. In vitro studies of the host-pathogen interaction exclude the inflammatory milieu, but a major challenge in studying the host response in vivo is dissecting pathogen-specific responses from the general inflammatory or common host response (Jenner and Young, 2005) that occurs as a result of tissue insult during the infectious process. This latter response is in part driven by activation of components of the complement cascade (Ricklin et al., 2010), the coagulation pathway (Oikonomopoulou et al., 2012), and the acute phase response (Suffredini et al., 1999), which all come into play within minutes of infection, and is compounded by rapid changes to the inflammatory cytokine and chemokine environment (Oghumu et al., 2010, Sadik and Luster, 2012, Soehnlein and Lindbom, 2010). Each of these stimuli may have profound effects on phagocyte function (Galvan et al., 2012, Hoffmann et al., 2010) and reflect, in the broader sense, the normal and/or essential response of phagocytes to inflammatory insult. Hence, responses that directly reflect intracellular infection are difficult to distinguish from dominant inflammatory signatures by conventional experimental approaches. We hypothesized that it might be possible to use the transcriptomic response of uninfected cells exposed to inflammation as a measure of the baseline or normal in vivo response to insult. Following this argument, transcriptomic networks specifically observed in infected cells, but not in neighboring uninfected cells, would represent cell-intrinsic pathogen-induced changes. In contrast, transcriptomic networks restricted to uninfected cells would serve to identify responses potentially beneficial to the host, the lack of which (in infected cells) might favor pathogen survival.

We tested the above hypothesis in a tractable experimental model of *Leishmania* infection, a pathogen associated with a significant global health burden (Alvar et al., 2012). *Leishmania* infect a variety of myeloid cells (including tissue-resident macrophages, inflammatory monocytes, dendritic cells [DCs], and neutrophils), but in models of experimental visceral leishmaniasis, *Leishmania donovani* amastigotes have a predilection for resident tissue macrophages, including Kupffer cells (KCs), the resident tissue macrophage lining the hepatic sinusoids (Beattie et al., 2010, Murray, 2001).

In this study, we discovered that expression of an *Rxra*-centered transcriptomic pathway, which is downregulated in KC in response to inflammation, is aberrantly maintained in infected cells. Pharmacological manipulation of RXRα activity perturbed the transcriptomic network of infected KCs, leading to enhanced leishmanicidal activity. This study demonstrates that aberrant expression of the *Rxra*-centered transcriptional pathway in *Leishmania*-infected KCs favors parasite survival and validates the use of cells exposed to inflammation as a means to discover pathogen-regulated transcriptomic pathways in vivo.

## Results

### An In Vivo Assay for the Analysis of Kupffer Cell Behavior

**Figure 1:**
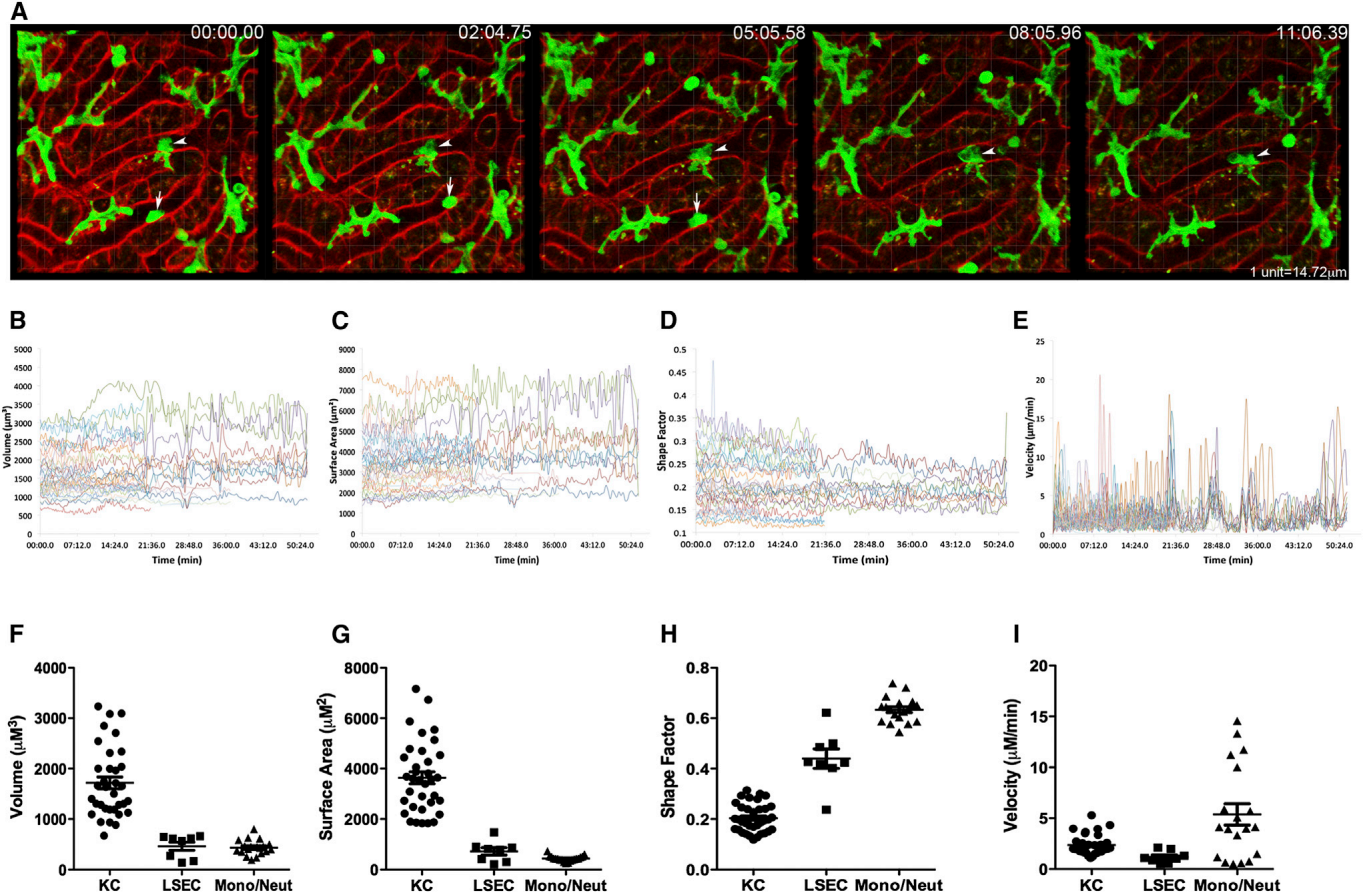
An In Vivo Assay for the Analysis of Kupffer Cell Behavior (A) Intravital analysis of KC (green) in liver sinusoids (red) in naive (LysM-Cre × mT/mG)_F1_ mice. Arrows indicate moving monocytes and/or neutrophils. Arrow heads show stationary KCs. See also Movie S1. (B–E) Measurement of the parameters of cell volume (B), surface area (C), shape factor (D), and velocity (E) for single KCs in 3 dimensions over time. Data represent 42 KCs from 2 mice. (F–I) Shown are the average volume (F), surface area (G), shape factor (H), and velocity (I) over time for KC, LSEC, and monocytes and/or neutrophils. Data are represented as mean ± SEM. Data represent 35 KCs, 8 LSECs, and 19 monocytes and/or neutrophils from 2 mice.

In the livers of uninfected (LysM-Cre × mT/mG)_F1_ mice, KCs were obvious from their large size, expression of membrane GFP, and their continued and intimate contact with liver sinusoidal endothelial cells, as has been previously described (Lee et al., 2010, Naito et al., 2004). Four-dimensional (4D) intravital two-photon imaging further allowed the fine membrane structure and lamellipodia dynamics of steady-state KCs to be observed in exceptional detail (Figure 1A; Movie S1). The continued extension and retraction of KC lamellipodia manifested itself as small fluctuations of cellular volume (Figure 1B) and surface area over time (Figure 1C). In contrast, monocytes and/or neutrophils were identified as moving rapidly through the sinusoidal spaces, whereas liver sinusoidal endothelial cells (LSECs) lined the sinusoidal lumen (Figures 1F and 1G). Shape factor, a quantitative measure of how close a cell is to a perfect sphere (shape factor = 1), showed small fluctuations over time for KCs (Figure 1D) but was always lower than that for either LSECs or monocytes and/or neutrophils (Figure 1H). The effects of KC membrane fluctuation can also be represented by a velocity, which was calculated by measuring the movement around the centroid point of the cell in three dimensions. KC velocity showed significant fluctuations over time (Figure 1E). Thus, although KCs do not actively migrate along the sinusoids, velocity as measured here provides an indication of the extent of membrane activity. In contrast, nonmotile LSECs have an even lower velocity (limited membrane activity). Many of the monocytes and/or neutrophils that were examined actively migrated along the sinusoids within the time frame of observation, and their velocity was not surprisingly high (Figure 1I).

### Phagocytosis of *L. donovani* Amastigotes Results in Rapid Activation of Both Infected and Uninfected KCs

**Figure 2:**
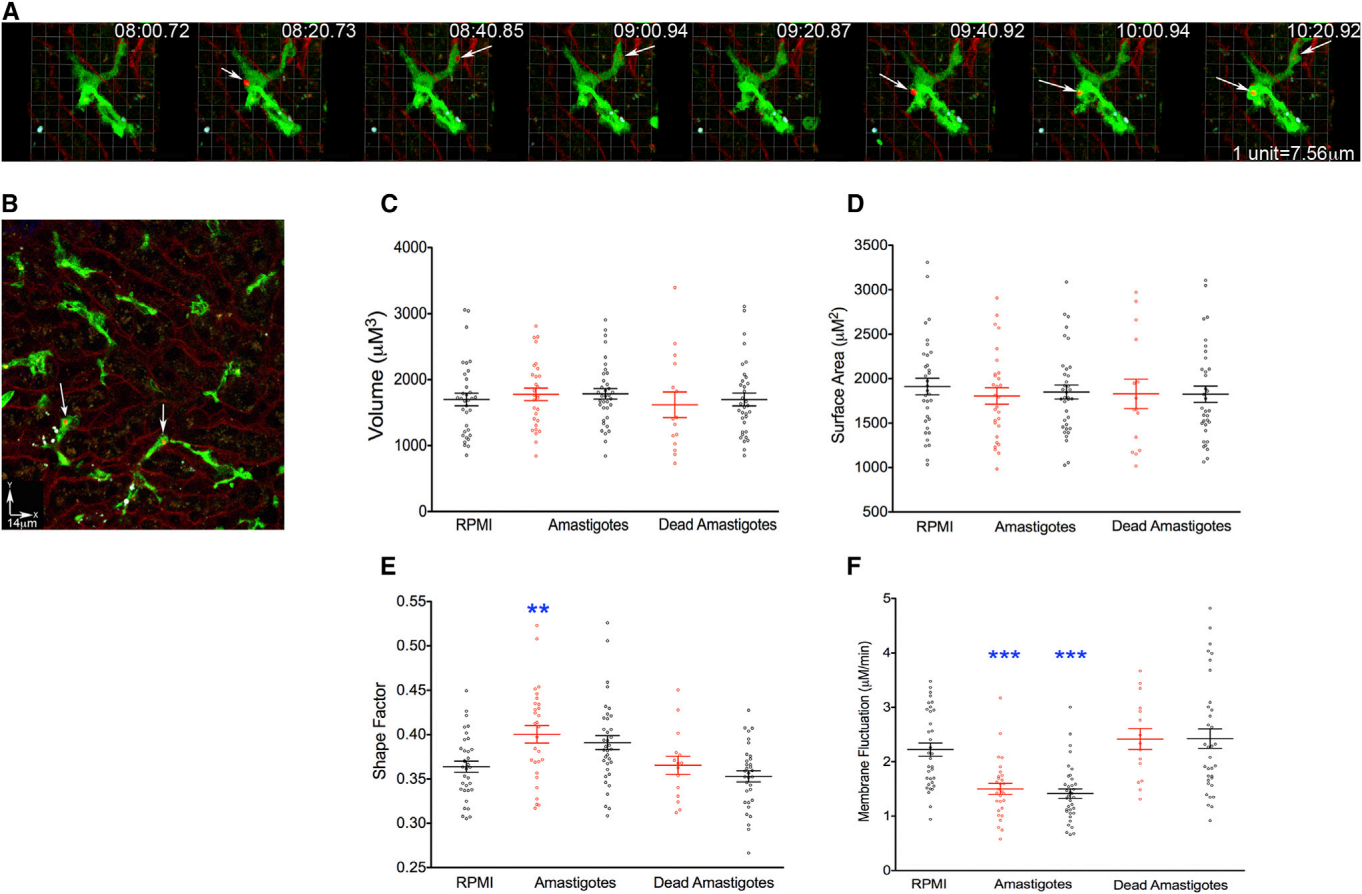
*Leishmania* Injection Causes Rapid Activation of Both Infected and Uninfected KCs (A) Real-time injection of *L. donovani* amastigotes (red, white arrows) and phagocytosis by KC (green). See also Movie S2. (B–F) Intravital imaging for 2 hr postinjection (B) and analysis of both infected (white arrows) and uninfected KCs for the parameters of volume (C), surface area (D), shape factor (E), and velocity (F). Infected KCs (red), inflamed KCs (black). Data are represented as mean ± SEM. Data represent 34 KC from 2 mice for RPMI, 29 infected KCs, and 36 uninfected KCs from 3 mice for live amastigotes, and 15 infected and 33 uninfected KC from 3 mice for dead amastigotes. Data were analyzed using a one-way ANOVA; ^∗∗^p < 0.01, ^∗∗∗^p < 0.001.

As the early response of KCs to infection with *L. donovani* amastigotes in vivo is largely uncharacterized, we examined KC behavior before and after infection in live mice. We injected (LysM-Cre × mT/mG)_F1_ mice with transgenic Td-Tomato-expressing *L. donovani* amastigotes (Beattie et al., 2010) at a dose that resulted in approximately 20% of KCs becoming infected over a 2 hr time period (a time by which most parasites have been cleared from the blood by KCs and other tissue phagocytes; Figures 2A and 2B; Gorak et al., 1998). The binding of amastigotes to the KC membrane (Figure 2A) was followed by complete phagocytosis within ∼20 s (Movie S2). Neither injection of live, nor of heat-killed, amastigotes resulted in detectable changes in KC volume (Figure 2C) or surface area (Figure 2D), irrespective of whether or not the KCs contained visible amastigotes. In contrast, injection of live, but not dead, amastigotes induced a rapid increase in the average shape factor that was significant for KCs that contained live parasites and showed a trend in this direction for KCs that did not contain amastigotes (Figure 2E). When velocity was measured as an indicator of membrane activity (Figure 2F), a significant decrease was observed in both infected and uninfected KCs following injection of live amastigotes, suggesting that both populations of cells were responding to the inoculated parasites through rapid activation. Such activation was not observed following injection of dead amastigotes (Figure 2F). Collectively, these data were indicative of a cellular response to active infection, rather than a response merely to nonspecific debris that may be present within the amastigote preparations. The observation that uninfected KCs in the immediate vicinity of infected cells also responded to the infection by loss of membrane activity (Figure 2F) suggested that the above changes in KC behavior were the result of either signals provided from infected KCs and operating rapidly in *trans* or from the activation of serum-derived mediators of inflammation, rather than being a direct consequence of intracellular parasitism per se. In either case, the capacity to activate this response was absent from heat-killed amastigote preparations.

### Analysis and Separation of Kupffer Cells Shows Heterogeneity in the Infected and Uninfected Populations

**Figure 3:**
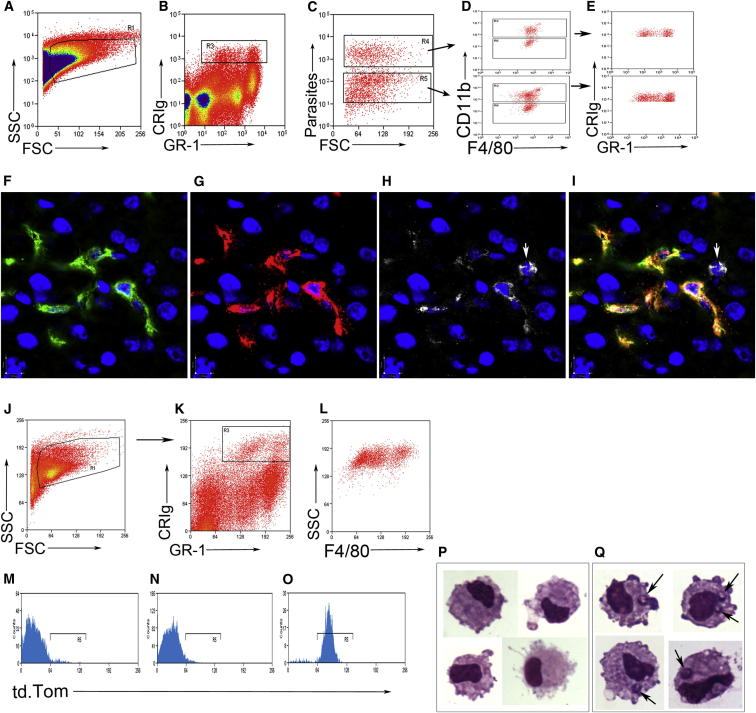
Analysis and Isolation of Infected and Inflamed KCs (A–C) C57BL/6 mice were infected with PKH26-labeled *Leishmania donovani* amastigotes, and the KCs were examined 12 hr later. KCs were gated on FSC and SSC (A), CRIg and GR-1 expression (B), and the presence of parasites (C). (D and E) Infected and uninfected KCs showed similar expression profiles for CD11b and F4/80 (D) and even distribution among the CRIg^+^ GR-1^lo^ and CRIg^+^ GR-1^hi^ populations (E). (F–I) Labeling of liver sections with F4/80 (F), CRIg (G), and GR-1 (H), or overlay of the three with DAPI (blue) (I), shows variable expression of GR-1 on KCs with a CRIg^−^ F4/80^−^ GR-1^+^ neutrophil noted (white arrow). (J–L) KCs were sorted 2 hr and 12 hr postinjection of tdTomato-*L. donovani* amastigotes, based on FSC and SSC (J), expression of CRIg and GR-1 (K), and expression of F4/80 (L). (M–O) The sorted KCs (M) were then further sorted into uninfected (N) and infected (O) populations. (P and Q) Giemsa-stained cytospins confirmed macrophage morphology and absence (P) or presence (Q) of amastigotes (arrows). See also Table S1.

As we wished to examine the transcriptional events that accompanied the observed change in KC behavior, we developed a method to identify and isolate KCs based on whether they contained parasites (herein termed infected) or not (herein termed inflamed). Flow cytometry analysis of hepatic mononuclear cell preparations with gating on forward and side scatter (Figure 3A), complement receptor of the immunologlobulin superfamily (CRIg) (Helmy et al., 2006), and Gr-1 (Figure 3B) demonstrated that both infected and inflamed populations of KCs could be observed and discriminated by the presence of fluorescent amastigotes (Figure 3C). Both infected and inflamed KCs showed heterogeneous expression of CD11b but relatively uniform expression of F4/80 (Figure 3D). Backgating on the infected and inflamed KCs showed that both populations contained both CRIg^+^ Gr-1^High^ cells and CRIg^+^ Gr-1^int^ cells (Figure 3E). To confirm that a proportion of KCs express Gr-1 in situ, we labeled liver sections with F4/80, CRIg, and Gr-1 (Figures 3F–3I) and observed variable expression of Gr-1 on KCs, in addition to GR-1^+^ neutrophils that did not express CRIg or F4/80 and were readily identifiable from their irregular-shaped nuclei (Figures 3H and 3I).

We then used fluorescence-activated high-speed cell sorting to isolate KCs and separate them into either infected or uninfected cell populations. We identified KCs from infected mice at 2 hr and 12 hr postinfection (p.i.), as described above, based on forward and side scatter (size and granularity) (Figure 3J) and expression of CRIg, GR-1 (Figure 3K), and F4/80 (Figure 3L) and then further sorted these based on whether or not they contained parasites (Figures 3M–3O). Cytospins of the sorted populations demonstrated that the cells had macrophage-like morphology, that neutrophils were not included in the analysis, and the presence of amastigotes within the infected population (Figure 3Q), but not in the inflamed population (Figure 3P). KCs were also isolated from sham-infected mice to serve as a control population that exhibited steady-state activity.

### Transcriptomic Profiling of Inflamed and Infected KCs Demonstrates Both Common and Distinct Pathways

To determine whether there were underlying transcriptional events that differed between KCs infected with amastigotes in an inflammatory setting and those induced in KCs exposed to inflammatory stimuli alone, transcriptomic profiles for each population, in addition to KCs from sham-infected controls, were generated by microarray analysis. Instead of performing a direct comparison of the differences in gene expression between the infected and inflamed KCs, we generated a list of genes differentially expressed between infected KCs and sham-infected KCs (using a cutoff of >3-fold difference). This approach eliminated the possibility that any of the regulated genes were induced or suppressed due to the isolation procedure, as the sham-infected KCs were subjected to the same digestion and sort process. The same analysis was then performed for the inflamed KCs. As a result of this analysis, we therefore generated two gene lists (infected and inflamed KCs) for 2 hr p.i. and two gene lists for 12 hr p.i. At 2 hr p.i., the expression of 1,188 genes (909 up and 279 down) was differentially regulated between sham-treated and infected KCs, whereas only 353 genes (327 up and 26 down) were differentially regulated between sham-treated and inflamed KCs. By 12 hr p.i., the number of genes showing changes in expression was substantially increased (2,135 between sham-treated and infected KCs, of which 1,337 had increased, and 798 had decreased, expression). STRING analysis of these genes showed that although there were some interactions between these genes, there were a large number of genes that were outside of the central network (Figure S1). Although substantially fewer genes (1,288) were differentially expressed at a messenger RNA (mRNA) level between the sham-treated and inflamed KCs overall (713 upregulated and 575 downregulated), STRING analysis for these genes showed a higher degree of connectivity between the genes within this list and a substantial network connection that did not appear to be present in the infected cells (Figure S1). The top 20 genes (based on fold change compared to sham-infected KCs for each comparison, or according to relative expression) are provided in Table S1. We interpret these data as indicating that there are genes that are normally regulated as part of the inflammatory response that are not regulated in infected cells.

### *Rxra* Forms the Hub of a Transcriptomic Network that Is Regulated in Inflamed, but Not Infected, KCs

**Figure 4:**
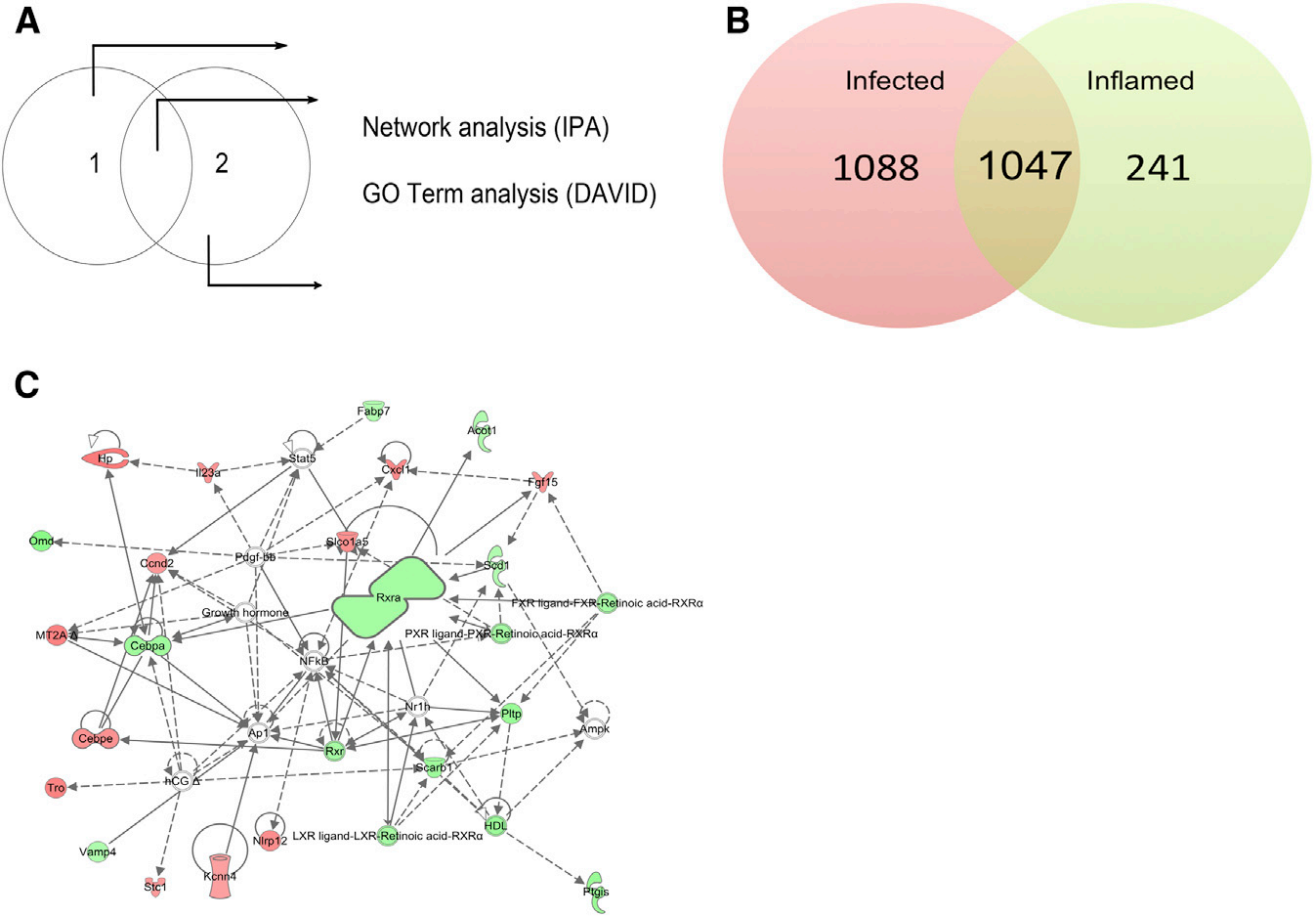
Comparative Subtraction Demonstrates Aberrant Expression of a Lipid Metabolism Pathway Centered around *Rxra* in Infected KCs (A) The method used for the separation and analysis of genes regulated in infected KCs, in inflamed KCs, or in both. (B) The number of genes in each list at 12 hr postinfection. (C) The lipid metabolism, small-molecule biochemistry, and molecular transport pathway. Green symbols denote downregulated genes, red symbols denote upregulated genes. Arrows with unbroken lines indicate a direct interaction between two molecules, with the mode of action in the direction of the arrow; arrows with broken lines denote an indirect interaction. See also Figure S1.

To determine the identity of genes and gene pathways that were common between the infected and inflamed cells, and those that were unique to either population, we performed a subtractive analysis of the gene lists (Figure 4A). From this analysis, we could readily identify genes that were either commonly regulated in both infected and inflamed cells or uniquely regulated in either infected or inflamed KCs at both 2 hr and 12 hr p.i. (Figures 4A, 4B, and S1C). For all genes identified as being selectively or commonly expressed in infected KCs and inflamed KCs, we have provided the top 20 genes ranked by expression level (Table S2). The gene lists were then subjected to pathway and gene ontology (GO) analysis (see Experimental Procedures for detail). A total of 1,088 genes were uniquely regulated in their expression at 12 hr p.i. in infected cells. Genes that showed differentially regulated expression in both infected and inflamed cells were enriched for GO terms suggestive of responsiveness to inflammation, including host defense and inflammatory response and cytokine-cytokine receptor interaction and chemokine activity.

Significantly, we identified 241 genes that were differentially regulated in their expression in inflamed cells, but not in cells that contained intracellular amastigotes, in spite of exposure of these cells to the same inflammatory insult. Ingenuity pathway analysis (IPA) was then used to identify genes that fell within IPA-generated pathways at a higher frequency than would normally be expected to occur for a randomly selected set of genes. This analysis identified 8 interacting networks within the inflamed gene list, in which genes involved in cell-to-cell signaling and the inflammatory response (IPA network score of 38), cell death (IPA network score of 37), antigen presentation (IPA network score of 35), cancer (IPA network score of 26), immune cell trafficking and cell cycle (IPA network score of 23), and cell morphology and cellular compromise (IPA network score of 10) were all overrepresented. The network that showed the highest level of gene enrichment was that of the lipid metabolism and small-molecule biochemistry pathway (Figure 4C), which had an IPA network score of 40. The fold change and the expression values for this pathway are shown in Table 1.

**Table 1:** Fold Change and Expression Values for Genes in the Lipid Metabolism and Small-Molecule Biochemistry Pathway, as Shown in Figure 4C

Gene Symbol	Fold Change Control versus Inflamed	Fold Change Control versus Infected	12 hr Control	12 hr Inflamed	12 hr Infected
*Omd*	−4.579	−2.848	0.76	−1.435	−0.75
*Ptgis*	−4.028	−2.937	0.577	−1.433	−0.977
*Pltp*	−3.998	−2.728	0.389	−1.61	−1.058
*Scarb1*	−3.819	−2.907	1.467	−0.466	−0.072
*Cepba*	−3.751	−2.479	0.864	−1.043	−0.446
*Rxra*	−3.56	−2.565	0.545	−1.286	−0.814
*Vamp4*	−3.164	−2.487	0.569	−1.092	−0.745
*Acot1*	−3.09	−1.512	−0.128	−1.756	−0.724
*Fabp7*	−3.085	−1.74	0.087	−1.538	−0.712
*Scd1*	−3.031	−2.183	0.399	−1.201	−0.728
*Kcnn4*	3.216	2.986	−0.617	1.069	0.962
*Stc1*	3.437	2.919	−0.857	0.924	0.689
*Ccnd2*	3.438	2.514	−0.491	1.291	0.84
*Fgf15*	3.573	1.846	−1.785	0.052	−0.901
*Il23a*	3.629	2.254	−1.027	0.832	0.145
*Cebpe*	3.714	2.821	−1.407	0.486	0.089
*Slcoa5*	3.786	2.448	−0.235	1.686	1.057
*Nlrp12*	3.842	1.901	−1.838	0.104	−0.911
*Tro*	4.198	2.356	−0.694	1.376	0.543
*Mt2a*	4.246	2.455	−1.307	0.779	−0.011
*Hp*	4.505	2.837	−1.944	0.227	−0.44

Table shows expression values (log2) for control, inflamed and infected cells, and fold change (positive values, upregulated; negative values, downregulated) in gene expression for inflamed and infected cells versus control cells. See also Table S2.

As the above networks were not regulated in infected cells, we hypothesized that they might represent genes and gene pathways for which homeostasis was favorable to parasite survival and, inter alia, that they represented genes potentially associated with antimicrobial defense regulated by inflammation. To test this hypothesis, we selected the most substantially represented network for further proof-of-concept studies (Figure 4C).

**Figure 5:**
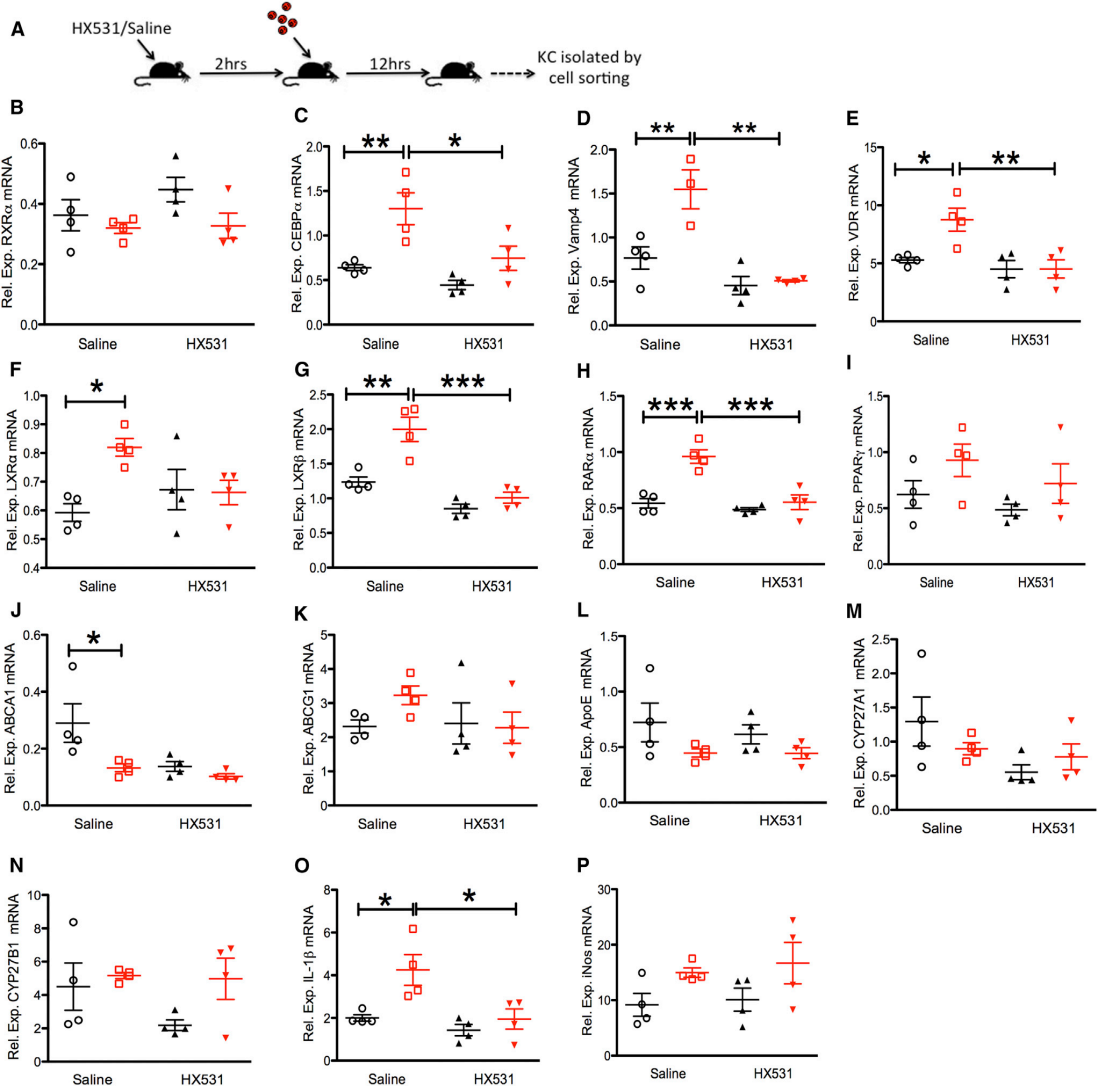
Inhibition of RXRα Modulates Gene Expression in Infected and Inflamed KCs (A) Mice were treated with HX531 or saline and then infected. (B–P) After 12 hr, infected (red) and inflamed (black) KCs were isolated to determine the relative mRNA accumulation of *Rxra* (B), *Cebpa* (C), *Vamp4* (D), *Vdr* (E), LXRα (F), LXRβ (G), *Rara* (H), *Pparγ* (I), *Abca1* (J), *Abcg1* (K), *ApoE* (L), *Cyp27a1* (M), *Cyp27b1* (N), *IL-1β* (O), and *iNos* (P) expressed as the relative expression compared to KC isolated from sham-infected control mice (relative expression of 1). Individual symbols represent cells isolated from pools of four mice, sorted independently. Data are represented as mean ± SEM. Data were analyzed using one-way ANOVA with Tukey’s post test. ^∗^p < 0.05, ^∗∗^p < 0.01, ^∗∗∗^p < 0.001. See also Figure S2.

IPA identified that, 11% (27/241 genes) of genes selectively regulated in their expression in inflamed KCs were found within the lipid metabolism and small-molecule biochemistry pathway (Figure 4C), which had retinoid X receptor α (*Rxra*) as a key hub (with *Rxra* itself being downregulated 3.5-fold in inflamed cells). We then sought to confirm the level of mRNA accumulation in infected and inflamed KCs in independent samples by quantitative real-time PCR (Figure 5). Although *Rxra* mRNA expression was not significantly different in infected and inflamed cells (Figure 5B), other genes within this network were validated as being differentially expressed between infected and inflamed cells, including *Cebpa* (Figure 5C), *Vamp4* (Figure 5D), LXRα/*Nr1h3* (Figure 5F), and LXRβ/*Nr1h2* (Figure 5G).

### Pharmacological Modulation of RXRα Activity In Vivo Inhibits the Upregulation of the *Rxra* Network-Associated Genes in Infected KCs

As infection with *Leishmania* resulted in maintenance or upregulation of expression of genes within the *Rxra*-associated gene network, we reasoned that if this was associated with parasite survival, then forced down modulation of RXRα activity might sufficiently perturb the entire *Rxra*-related gene network within infected cells to generate an enhanced antimicrobial effect. We therefore tested this approach by targeted pharmacological intervention, using a small-molecule antagonist HX531, specific for RXRα (Ebisawa et al., 1999, Yamauchi et al., 2001). To first establish that HX531 treatment had a direct effect on the gene network in infected KCs, we treated mice with HX531 or saline, infected the mice with *L.donovani*, and isolated infected or inflamed KCs to examine gene expression by quantitative real-time PCR (Figure 5A). HX531 treatment had no significant effect on the expression of *Rxra* at an mRNA level (Figure 5B), but treatment resulted in a significant modulation of the expression of a number of network-associated genes, including *Cebpa* (Figure 5C), *Vamp4* (Figure 5D), *Vdr* (Figure 5E), LXRβ (but not LXRα) (Figures 5F and 5G), and *Rara* (Figure 5H). Importantly, this effect was restricted to infected KCs. Peroxisome proliferator-activated receptor γ (*Pparγ*) was not regulated in infected cells (Figure 5I) and showed no change in expression following HX531 treatment. We also examined the effect of HX531 treatment on a number of RXR-related genes that were not represented within the identified network (Figure 4C), including *Abca1* (Figure 5J), *Abcg1* (Figure 5K), *ApoE* (Figure 5L), *Cyp27a1* (Figure 5M), and *Cyp27b1* (Figure 5N). HX531 treatment had no measurable effect on the expression of these genes in KCs, with the exception of *Abca1*, where treatment appeared to block upregulation in uninfected cells (Figure 5J). HX531 treatment blocked the upregulation of *IL-1β* in infected KCs (Figure 5O) but had no effect on inducible nitric oxide synthase (iNOS) mRNA levels in infected or inflamed KCs (Figure 5P).

To confirm that genes within this network were not subject to regulation merely by phagocytosis, we performed RT-PCR for a subset of genes within this network on KC isolated from mice injected with latex beads. As shown in Figure S2, uptake of latex did not affect mRNA levels for *Rxra* (Figure S2A), LXRβ (Figure S2B), *Cebpa* (Figure S2C), or *Vamp4* (Figure S2D), relative to KC isolated from control-injected mice.

### Pharmacological Modulation of RXRα Activity Reduces *Leishmania* Survival in KC

**Figure 6:**
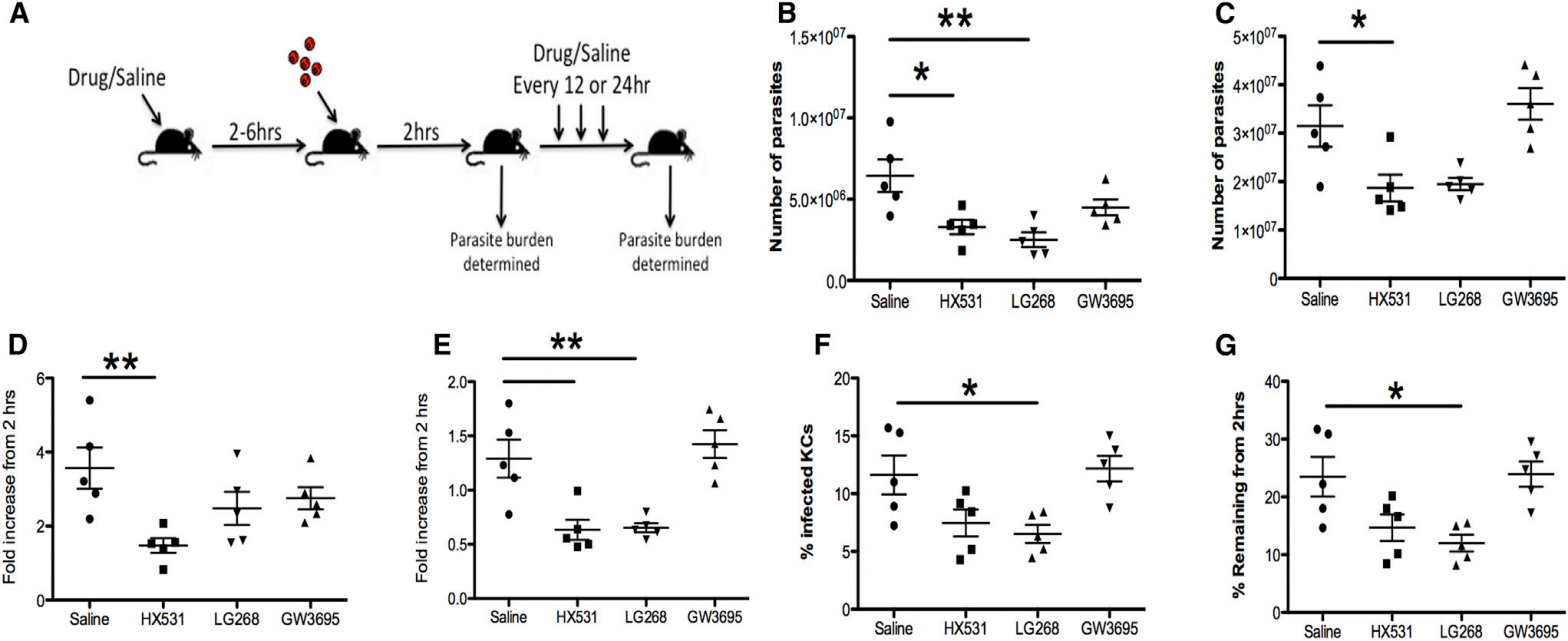
Therapeutic Targeting of RXRα Impairs *L. donovani* Survival within Macrophages (A) Mice were pretreated with HX531, LG268, GW3965, or saline and infected 2–6 hr later. Parasite burdens were determined after 2 hr and 96 hr. (B and C) Number of parasites present in the spleen (B) and liver (C) 96 hr after infection, as measured by flow cytometry. (D and E) The fold increase in parasite burden relative to the 2 hr time point in the spleen (D) and liver (E). (F) The percentage of KCs infected at 96 hr determined by fluorescence microscopy. (G) The percentage of KCs infected at 96 hr relative to the percent infected at 2 hr. Individual symbols represent individual mice, and data are representative of three independent experiments for HX531 treatment and control groups and 1 experiment for LG268 and GW3965 treatments. Data are represented as mean ± SEM.

To determine the effect that the perturbation of the RXRα pathway would have on infection, and hence to examine the potential of the expression profiling approach that we have used for the discovery of important host response pathways, we treated mice with HX351 prior to and for 4 days following infection with Td-tomato *L. donovani* amastigotes (Figure 6A). Using a flow cytometric approach to quantify the number of parasites in the tissues, we first assessed splenic and hepatic parasite burden at 2 hr p.i., when there was no difference in the presence or absence of treatment, and at 96 hr p.i. (Figures 6B and 6C). HX531 treatment significantly reduced the number of parasites present in both the liver and the spleen at 4 days postinfection relative to the saline-treated control animals. To examine whether perturbation in this pathway could also be achieved by stimulating, rather than inhibiting, RXRα, we treated mice with LG268 (an RXR agonist). LG268 also reduced the number of parasites present in the spleen but not the liver. To determine whether RXRα perturbation could be operating through effects on LXR, we treated mice with GW3965 (an LXR agonist). GW9395 treatment had no effect on parasite burden in either tissue (Figures 6B and 6C), suggesting that LXR is not involved in control of parasite load mediated through the RXRα network. In order to examine if the therapeutic effect observed reflected leishmanistatic or leishmanicidal activity, we also calculated the fold change in parasite numbers in the 2 organs at 4 days p.i. relative to the number of parasites present at 2 hr p.i. (Figures 6D and 6E). In spleens of untreated mice, parasite numbers increased over a 96 hr period, and this growth was inhibited after drug treatment. In contrast, in liver, parasite numbers did not increase significantly over 96 hr in nontreated mice and were reduced to below the level seen at 2 hr p.i. in drug-treated mice, suggestive of leishmanicidal activity (Figure 6E). To confirm this observation, we used a histological approach in the liver and further showed that the percentage of infected KCs was significantly reduced in the LG268-treated animals and showed a trend in this direction in HX531-treated mice but not in GW3965-treated mice (Figures 6F and 6G). Although not significant in the single experiment shown in Figure 6, replicate experiments with mice treated with HX531 confirmed that this drug enhanced the leishmanicidal activity of KCs (p = 0.0028; n = 11 mice for saline controls and 8 mice for HX531 treatment). In conclusion, these data indicate that pharmacological manipulation of RXRα function was sufficient to perturb the gene network that we identified as being aberrantly expressed in infected KCs. Furthermore, it was unlikely that the effects of HX531 or LG268 were due to direct effects on LXR, as stimulation of LXR had no measurable biological effect.

## Discussion

We have examined the early in vivo response of liver macrophages to infection with *Leishmania*, independently studying the activation of infected and uninfected cells exposed to inflammatory signals within the infected tissue microenvironment. Our approach provides a number of unique insights into the transcriptomic response induced by *L. donovani* infection in vivo and provides proof of concept that the use of cells exposed to inflammation is a powerful method for the discovery of pathogen-regulated gene pathways.

First, the isolation of KC from infected mice revealed a transcriptional response in infected KCs that is somewhat distinct from that observed by others using different types of mononuclear host cells (including bone marrow-derived macrophages, monocytes, and DCs). It remains to be determined whether this reflects cell specificity in host response or differences due to the analysis being performed on cells infected in vitro versus in vivo. In most previous in vitro experiments, the possible influence of uninfected cells on gene expression profile, e.g., by dilution of the signal associated with infected cells, was not taken into account (Buates and Matlashewski, 2001, Chaussabel et al., 2003, Osorio y Fortéa et al., 2009, Rodriguez et al., 2004). In one series of studies, DCs infected in vitro with *L. amazonensis* were sorted to allow microarrays to be conducted only on infected cells (Giraud et al., 2012, Lecoeur et al., 2010). While these studies revealed unique signatures not evident from bulk population analysis, data were not provided to show whether the uninfected DC within the culture system had also made bystander responses (e.g., mediated by cytokine release from infected DCs in culture). Furthermore, the transcriptomic signatures generated by microarray in this and indeed most other studies (including those cited above) have not been validated fully at the protein level.

Second, our data demonstrate significant quantitative and qualitative differences in transcriptomic responses compared to previous in vitro gene expression studies. In vitro experiments have usually been interpreted as indicating that visceralizing species of *Leishmania* result in a relatively silent infection of macrophages (Zhang et al., 2010), significant suppression of gene expression (Buates and Matlashewski, 2001), or a signature distinct from that associated with interferon (IFN)-γ activation (Rodriguez et al., 2004). The tendency of *L. donovani* to induce a weak inflammatory response in vitro may be species specific, with *L. donovani* metacyclics inducing lower fold changes in inflammatory gene expression compared to *L. major* metacyclics in one study (Chaussabel et al., 2003). Alternatively, there may be life cycle stage-specific determinants of gene expression, as shown for *L. mexicana* (Aebischer et al., 2005). In an additional study, *L. amazonensis* amastigotes were also shown to induce significant gene expression changes in vitro, including changes to the sterol biosynthesis pathway (Osorio y Fortéa et al., 2009). Importantly, the impact of *L. chagasi* on host macrophage response was significantly modulated by the addition of T cells to the culture in vitro (Ettinger and Wilson, 2008), suggesting that factors external to the infected macrophage impact the response of the infected cell. Our data support and extend these observations by adding the full in vivo complexity of the microenvironment created in the infected liver.

Third, we show that uninfected KCs also undergo significant biological and gene transcriptional changes associated with the inoculation into mice of live *L. donovani* amastigotes. The activation of uninfected KCs at early time points, with further downstream transcriptional consequences seen at 12 hr, suggests that soluble mediators or signals operating in *trans* facilitate global activation of macrophages within the infected tissue. Although this activation shows both qualitative and quantitative differences between infected and inflamed KCs, many of the genes that have been associated with cell-autonomous defense against intracellular protozoa stimulated by interferons (MacMicking, 2012) are induced to similar levels in infected and uninfected KCs at 12 hr postinfection. This would place such genes in the category of commonly-regulated genes within the framework of our analysis. Such genes include the guanylate-binding proteins *Gbp2* and *Gbp5*, indoleamine 2,3-dioxygenase 1 (*Ido1*), immunity-related GTPase family M members 1 and 2 (*Irgm1* and *Irgm2*), and immunoresponsive gene 1 (*Irg1*). These data are somewhat intuitive, given that IFN is a soluble mediator, capable of *trans*-activating neighboring cells.

Collectively, our comparative analysis of the gene expression profiles of infected and inflamed KCs taken from the livers of infected mice suggests that a degree of caution should be exercised when interpreting whether genes observed to alter their expression in response to infection truly represent autonomously regulated genes associated with the presence of intracellular pathogens.

Fourth, we have identified and validated the importance of a gene network centered around *Rxra* that is associated with the survival of amastigotes within the liver of *L. donovani*-infected mice. Within this *Rxra* network, we identified a predominance of genes associated with lipid metabolism. Although lipid metabolism centered around RXRα has not previously been implicated in *L. donovani* pathogenesis in mice, other studies have observed changes in lipid metabolism genes associated with *Leishmania* infection. For example, changes in components of the sterol biosynthesis pathway were induced by macrophage infection with *L. amazonensis* (Osorio y Fortéa et al., 2009). Previous studies have characterized a role for two heterodimeric partners of RXR in the control of *Leishmania major* infection, the vitamin D receptor (VDR) (Ehrchen et al., 2007, Whitcomb et al., 2012) and the liver X receptor (LXR) (Bruhn et al., 2010), with deficiency in either receptor resulting in enhanced resistance to infection. In addition, upregulation of VDR was previously reported following in vitro infection of macrophages with *L. chagasi* (Rodriguez et al., 2004) as well as *L. donovani* and *L. major* (Chaussabel et al., 2003), suggesting that VDR is an important component of the host response to *Leishmania* infection. We believe that by targeting RXRα as the hub of the identified network, we have successfully perturbed an entire gene network that controls *L. donovani* survival in KCs. Perturbation of RXR in either direction via activation with the agonist LG268 or suppression with the antagonist HX531 had similar effects on the growth of *L. donovani* in vivo. This suggests that a delicate balance of expression exists across the RXR gene network. The mechanism by which this network controls parasite survival is currently subject to further investigation. However, we speculate that the gene network may regulate the usage of lipids and cholesterol by the parasite while it is present in the phagolysosome of the host cell (McConville and Naderer, 2011). Alternatively, macrophage sensing of *Leishmania*-derived lipids may be having profound effects on the nature of the innate immune response that is initiated (Nagy et al., 2012).

Finally, and of broader significance, we have described an approach to study host-pathogen interactions, which is capable of revealing important host response pathways that would be invisible in studies of the interaction between macrophages and pathogens in vitro. Importantly, we show that manipulation of such pathways in vivo can impact parasite survival, reducing parasite load at early stages after liver infection. Targeting of nuclear receptors for therapy is a burgeoning field of medicine, with RXR therapeutics showing efficacy in clinical settings of cutaneous T cell lymphoma (Duvic et al., 2001, Khuri et al., 2001) and a preclinical model of Alzheimer’s disease (Cramer et al., 2012). RXR antagonists, including HX531, have also shown efficacy in preclinical models of diet-induced obesity and type 2 diabetes (Yamauchi et al., 2001). In this respect, while it is tempting to suggest that our findings may also set the foundation for the use of RXR-mediated therapies for leishmaniasis, detailed investigations into the mechanism of parasite growth arrest and/or killing are now required.

## Experimental Procedures

### Mice and Infection

C57BL/6 mice were obtained from Charles River (UK). mT/mG (Muzumdar et al., 2007) and LysMcre (Clausen et al., 1999) mice have been previously described. Mice were bred and housed under specific pathogen-free conditions and used at 6–12 weeks of age. The Ethiopian strain of *Leishmania donovani* (LV9) and tandem Tomato fluorescent protein expressing LV9 (tdTom.LV9) (Beattie et al., 2010) were maintained by serial passage in Rag-1^−/−^ mice. Amastigotes were isolated from infected spleens (Smelt et al., 1997), and mice were infected with 3 × 10^7^ *L. donovani* amastigotes intravenously (i.v.) via the tail vein in 200 μl of RPMI 1640 (GIBCO). Parasites were killed via heat treatment at 56°C for 15 min, followed by labeling with PKH26 Red Fluorescent Cell Linker Kit (Sigma). For latex bead experiments, 1 μm Fluoresbrite Polychromatic Red Microbeads (Polysciences) were washed twice in saline, and 3 × 10^7^ beads per mouse were injected i.v. All experiments were approved by the University of York Animal Procedures and Ethics Committee and performed under UK Home Office license (Immunity and Immunopathology of Leishmaniasis Ref # PPL 60/3708).

### Intravital Imaging

Mice were anesthetized, and surgery was performed similar to that previously described (Beattie et al., 2010), except that anesthesia was maintained by inhalation of 4% isofluorane (Abbott Laboratories). Images were acquired on an inverted LSM 780 Multiphoton Microscope (Zeiss) maintained at 36°C by a blacked-out environmental chamber (Solent Scientific). Images were acquired with a 40× 1.1 water immersion objective and fluorescence excitation provided by a Chameleon XR Ti:Sapphire Laser (Coherent) tuned to 870 nm. For 4D analysis, 20–35 μM z stacks were acquired with a z distance of 2 μM approximately every 15–30 s. Real-time injection was performed via tail vein catheter (Strategic Applications Incorporated, Infusion Technologies). In addition to collecting baseline data for animals before infection, individual KCs were imaged prior to and for 2 hr directly following infection of mice with *L. donovani*. Data were rendered and analyzed using Volocity software (PerkinElmer), and cell analysis was performed automatically with manual checking.

### Confocal Microscopy

Confocal microscopy was performed on 20 μm frozen sections as previously described (Beattie et al., 2011). Antibodies were conjugated to Alexa 488 or Alexa 647 (eBioscience). Slides were blinded before imaging on a Zeiss LSM 510 Axioplan Microscope (Zeiss). Data were rendered and analyzed using Volocity Software (Improvision).

### Flow Cytometry and Cell Sorting

Hepatic mononuclear cells were prepared from the livers of tdTom.LV9- or RPMI-injected C57BL/6 mice. A total of 64 mice were used for the microarray study. We conducted four independent infection experiments to generate samples for analysis. In each experiment, n = 16 mice, and at each time point studied (2 hr and 12 hr postinfection), we isolated KCs from the livers of four mice treated with RPMI and four mice infected with *L. donovani*. Liver mononuclear cells were pooled prior to sorting. For real-time PCR analysis, replicates represent pooled samples from independently sorted samples. Livers were perfused with 0.4 mg/mL of warmed Liberase TL (Roche) then digested at 37°C for 45 min. Digested livers were passed over 100 μM cell strainers, washed in PBS containing 2% fetal calf serum (FCS), and resuspended in 33% Percoll for centrifugation at 693 × *g* for 12 min. Isolated cells were blocked with anti CD16/32 then labeled with F4/80-Alexa 488, GR-1 PeCy7 (eBioscience), and CRIg (Helmy et al., 2006) that was conjugated to Atto 647H via lightning-link conjugation (Innova Biosciences). Cells were sorted on a MoFlo Cell Sorter (Beckman Coulter). Sorted cells were pelleted and resuspended in TRIzol (Invitrogen). A small fraction of cells (approximately 3,000 sorted cells) were spun onto glass slides, fixed in methanol, and stained with Giemsa for morphological analysis.

### Whole-Genome Array

RNA was isolated from purified KC and amplified via Agilent Low Input Quick Amp Labeling Kit (Agilent Technologies). Amplified RNA was then assayed with Agilent SurePrint G3 Mouse Gene Expression 8×60K Microarray Chips that were scanned with an Agilent C Scanner with SureScan High-Resolution Technology (Agilent). The data were normalized using the percentile shift method to the 80^th^ percentile. Comparison of the gene expression data between the RPMI controls and the infected or inflamed samples was performed using the Benjamini and Hochberg false discovery rate (FDR) correction (Pawitan et al., 2005a), albeit with the caveat that this approach may have low sensitivity with the sample size used (Pawitan et al., 2005b). This analysis was performed with GeneSpring software (version 9; Agilent) as a standard 5% FDR, with the variances assessed by the software for each t test performed. A 3-fold expression criteria was then applied to each gene list. All data for long intergenic noncoding RNA (lincRNA) probe sets were removed from the analysis at this point. Gene ontology analysis was performed using the DAVID (Huang et al., 2009) and Ingenuity Pathway Analysis software packages (Ingenuity Systems), and gene maps generated with STRING (www.string-db.org).

### Drug Treatment

Mice were pretreated with saline, 100 mg/kg HX531 (Yamauchi et al., 2001), 30 mg/kg LG268 (Shen et al., 2004), or 20 mg/kg GW3965 (Lei et al., 2013) 2–6 hr prior to infection and then every 24 hr until 72 hr for HX531 and LG268 or every 12 hr for GW3965. At 96 hr postinfection, the livers and spleens were collected, and the parasite burden was determined both by flow cytometry and in fixed liver slices. Data were normalized to the baseline 2 hr time point to determine the survival of parasites relative to the initial dose of infection.

### Determination of Parasite Burden by Flow Cytometry

Liver and spleens were collected, homogenized, and resuspended in a specific volume of RPMI 1640 (GIBCO). A total of 200 μl of each sample was aliquoted in duplicate to a 96-well plate, centrifuged at 2,000 × *g* for 10 min, and the pellets were resuspended in 2 mg/mL saponin for 5 min at room temperature (RT). The centrifugation was repeated and followed by resuspension in 1 mg/mL saponin. The remaining pellets were washed a further two times, and the wells were resuspended in 200 μl then spiked with 2 μl of 6 μM polystyrene microspheres (Polysciences, Inc.) at 4.6 × 10^5^ particles/μL. Samples were acquired on a CyAn ADP analyzer (Beckman Coulter) in which both forward scatter detector neutral density filters had been removed to allow for small-particle detection. The number of parasites was quantitated by determining the ratio of beads to parasites and then multiplying to calculate the total number of parasites in the starting volume.

### Real-Time PCR

Isolated cells were resuspended and frozen in QIAzol lysis buffer. RNA was isolated from sorted cell populations using a miRNeasy Kit (QIAGEN). RNA was reverse transcribed into complementary DNA (cDNA) with a Superscript III First-Strand Synthesis Kit (Invitrogen). Oligonucleotides used for the specific amplification of target genes are described in the Supplemental Experimental Procedures. Real-time quantitative PCR was performed with Fast SYBR Green PCR Master Mix in a StepOnePlus Real-Time PCR System (Applied Biosystems). Accumulation of target genes was normalized to HPRT, β-actin (Shih et al., 2012), or ribosomal protein L32 (Reis et al., 2013) and expressed as relative expression via the change in cycle threshold (ΔΔCT) analysis method (relative expression in test sample versus RPMI controls).

## Accession Numbers

The EBI ArrayExpress accession number for the microarray data reported in this paper is E-MEXP-3877.

## Supplemental Information

Document S1. Supplemental Experimental Procedures and Figures S1 and S2

Table S1. Related to Figure 3 The top 20 up- and downregulated genes for infected versus sham-infected or inflamed versus sham-infected KCs at 2 hr and 12 hr postinfection, ranked by fold change (sheet 1) or ranked by relative expression at 12 hr postinfection (sheet 2).

Table S2. Related to Table 1 Top 20 genes expressed selectively by infected KCs, selectively by inflamed KCs, or by both infected and inflamed KCs at 12 hr postinfection, ranked by expression values (log 2) in infected KCs (or expression in inflamed KCs for inflamed KCs only).

Movie S1. Related to Figure 1 Intravital analysis of KCs (green) in liver sinusoids (red) in naive _F1_(mT/mG × LysMcre) mice demonstrating rapidly moving neutrophils and/or monocytes and stationary, but active, KCs.

Movie S2. Related to Figure 2 Intravital analysis of phagocytosis of *L. donovani* amastigotes (red) by KCs (green) in real time.
