# Supplementary material for: A Transcriptomic Network Identified in Uninfected Macrophages Responding to Inflammation Controls Intracellular Pathogen Survival
Source: Cell Host Microbe. 2013 Sep 11;14(3):357–68. doi: 10.1016/j.chom.2013.08.004 (PMC4180915; doi:10.1016/j.chom.2013.08.004)
Supplement: Document S1. Supplemental Experimental Procedures and Figures S1 and S2 [file mmc1.pdf]

Cell Host & Microbe, Volume *14*

Supplemental Information

A Transcriptomic Network Identified in Uninfected Macrophages Responding to Inflammation Controls Intracellular Pathogen Survival

Lynette Beattie, Micely Del-Rei Hermida, John W.J. Moore, Asher Maroof, Najmeeyah Brown, Dimitris Lagos, and Paul M. Kaye

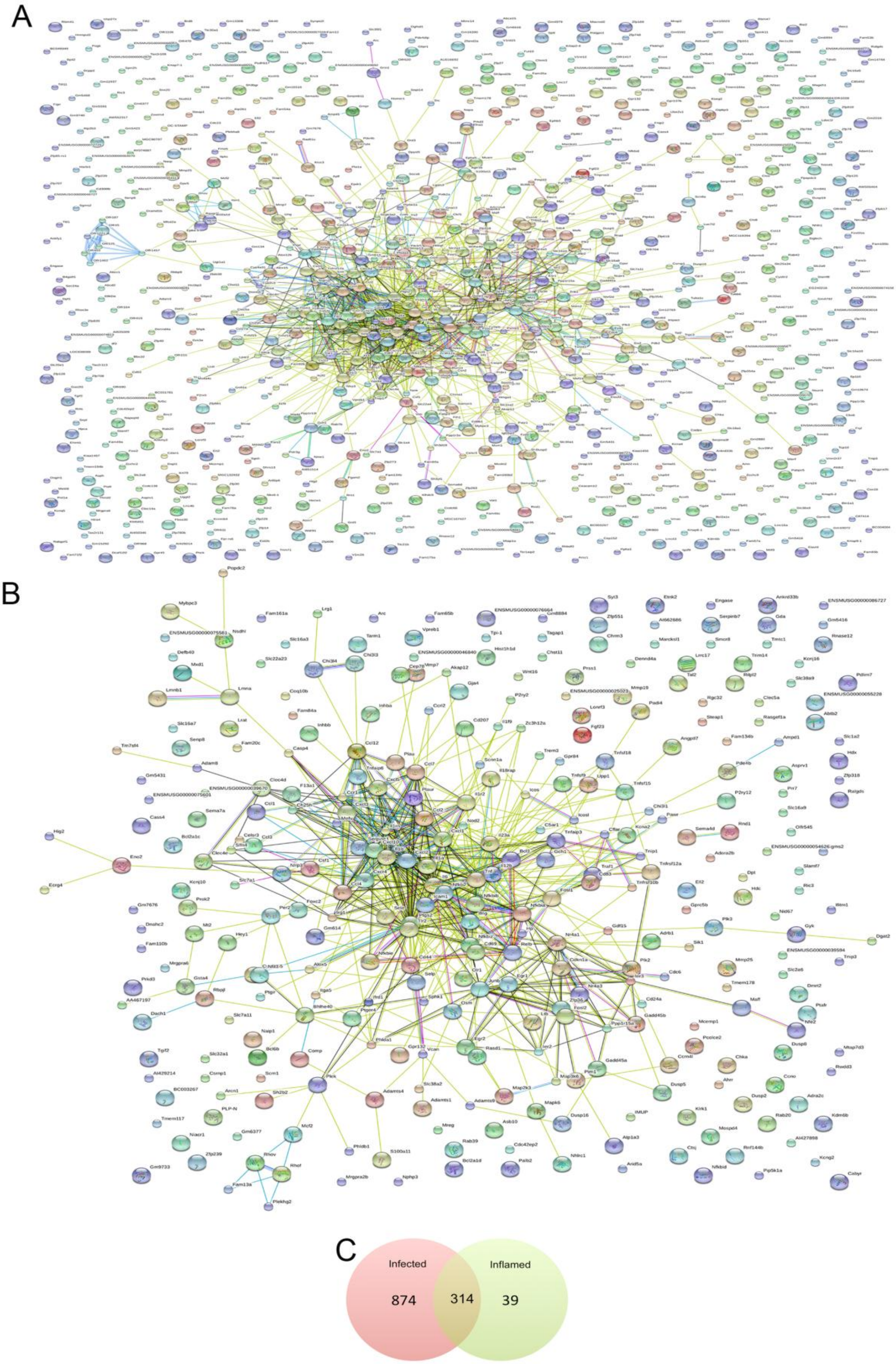
A
B
C
Infected
Inflamed
874
314
39

D

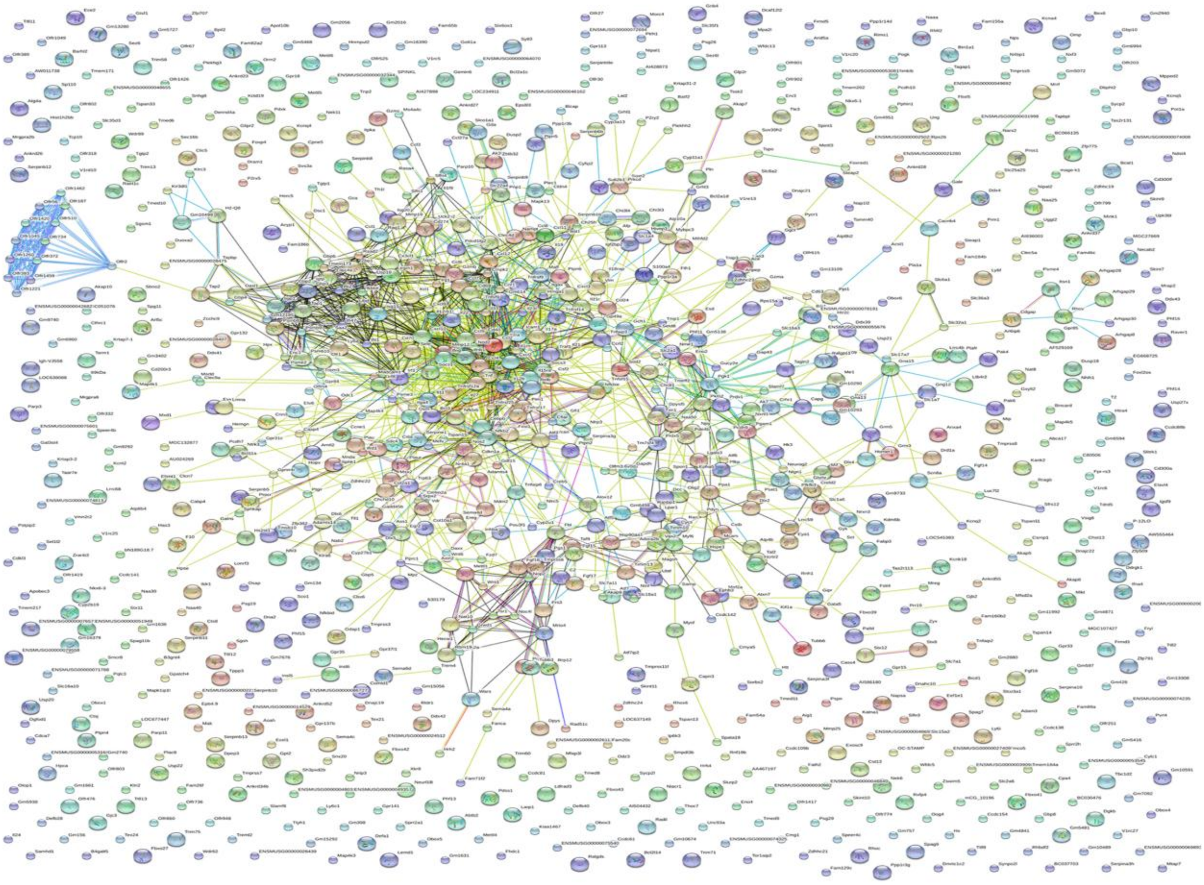

E

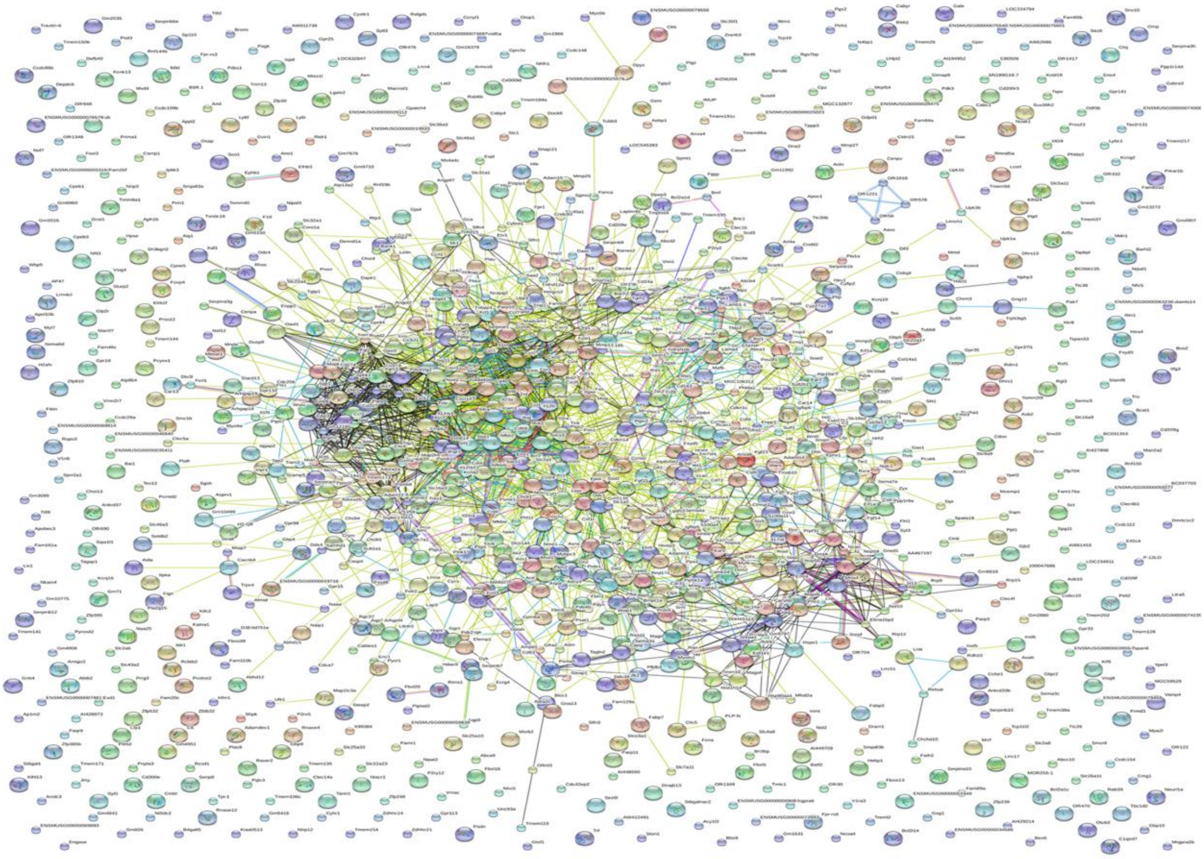

Figure S1. Comparison of Genes with Regulated Levels of Expression at 2 and 12 h Postinfection with *L. donovani*, Related to Figure 4

A) STRING analysis of the genes whose expression was 3 fold up or down regulated in infected KCs compared to control KCs or B) bystander activated KCs compared to control KCs at 2 hours post-injection of tdTomato-*L. donovani* amastigotes. Larger icons have a protein structure associated with them. This can be either a Protein Data Bank (PDB) entry for the protein itself or a close homolog. A small bubble (without icon) means that there is no structural information available. Nodes are either colored (if they are directly linked to neighboring nodes) or white (nodes of a higher iteration/depth). Lines are colored according to the type of evidence they represent. Green lines connect nodes that show immediate neighborhood on the genome (within 300 bp on the same strand). Red lines represent gene fusion. Blue lines represent co-occurrence. Black lines represent co-expression. Pink lines show nodes that are connected via direct experimental evidence. Turquoise lines represent genes that have interactions based on data mining from the databases: Biocarta, BioCyc, GO, KEGG, or Reactome. Light green lines represent text mining associations due to proteins being mentioned with other proteins in publications. Purple lines represent homology. C) The number of genes identified within each list at 2 hours post-injection. D) STRING analysis of the genes that were 3 fold up or down regulated in infected KCs compared to control KCs or E) inflamed KCs compared to control KCs 12 hours post-injection of tdTomato-*L. donovani* amastigotes.

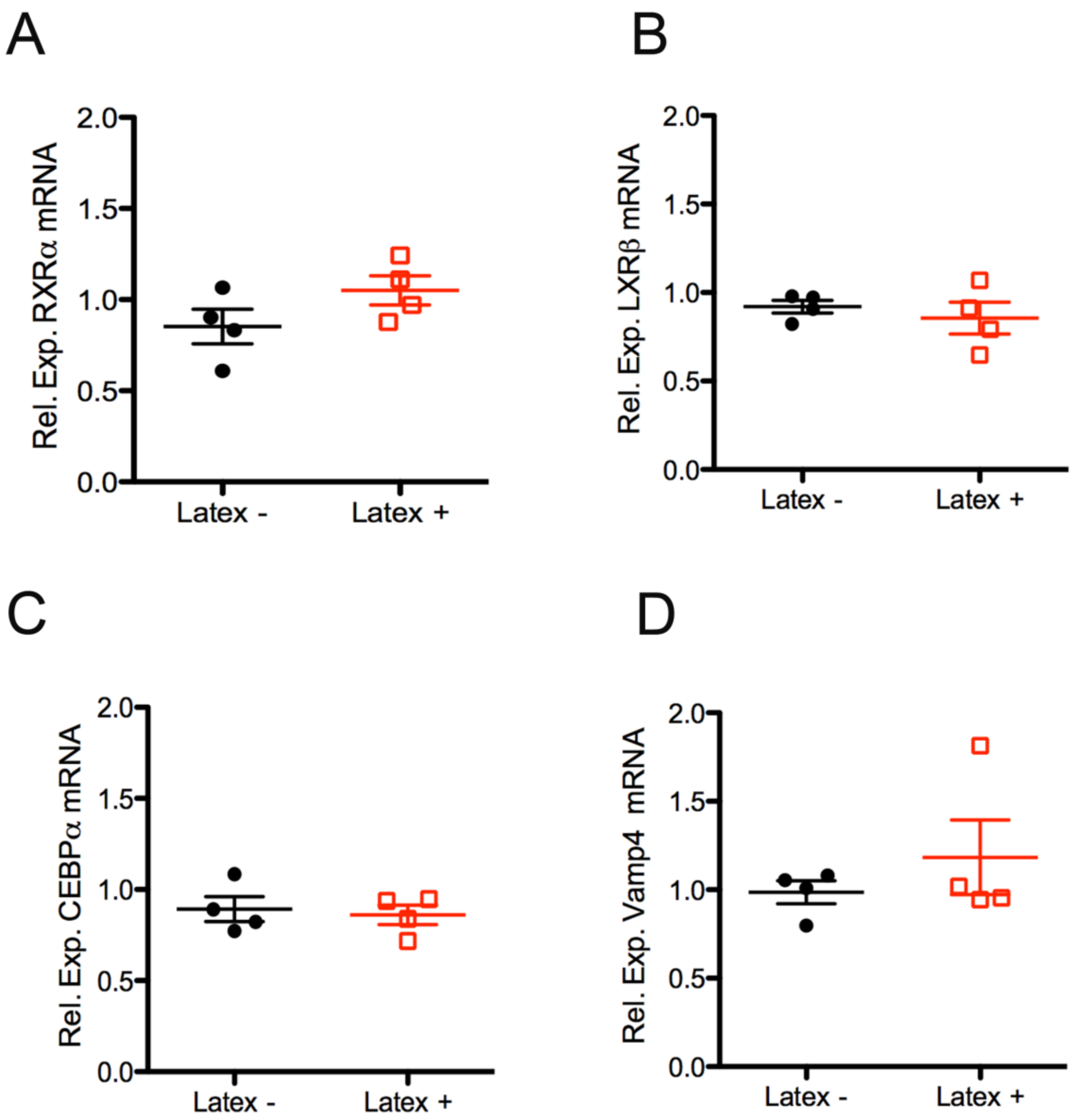

Figure S2. Gene Expression in KCs Following Latex Bead Phagocytosis, Related to Figure 5

A) Mice were injected with $3x10^7$ latex beads and KC isolated after 12 hours. mRNA abundance of *Rxrα*, B) *LXRβ*, C) *Cebpα* and D) *Vamp4* was determined relative to KC isolated from control injected animals (relative expression of 1). Black symbols represent KC that had not phagocytosed particles, red symbols represent KC that had phagoctyosed particles. Individual symbols represent cells isolated from pools of 4 mice, sorted independently. Data were analysed by one-way ANOVA with a Tukey post-test. Data are represented as mean +/- SEM.

Supplemental Experimental Procedures:

Primers Used for Real-Time PCR:

Oligonucleotides used for the specific amplification of target genes were either previously published or designed by qPrimerDepot (http://mouseprimerdepot.nci.nih.gov). All primers were verified to have a similar amplification profile as HPRT in a serial dilution series and a single peak in a melting curve analysis.

RXRα: AACCCCAGCTCACCAAATGACC/AACAGGACAATGGCTCGCAGG
CEBPα: TGGACAAGAACAGCAACGAGTA/GCAGTTGCCATGGCCTTGA
VDR:
CTCCTCGATGCCCACCACAAGACCTACG/GTGGGGCAGCATGGAGAGCGGAGACAG
LXRα: GGGAGGAGTGTGTGCTGTCAG/GAGCGCCTGTTACACTGTTGC
LXRβ: GGCCTGGACGATGCAGAGT/CGATCGGCTGAGAAGATGTTG
RARα:GGGAGGGCTGGGTACTATCT/AGCACCAGCTTCCAGTCAGT
PPARγ: GAGTGTGACGACAAGATTTG/GGTGGGCCAGAATGGCATCT
IL-1β: AACCTGCTGGTGTGTGACGTTC/CAGCACGAGGCTTTTTTGTTGT
iNos: CCCTTCCGAAGTTTCTGGCAGCAGCAGC/GGCTGTCAGAGCCTCGTGGCTTTGG
Vamp4: GGAGAAAGGGTAGCACACATCAG/CCACATGCCATACAGAACAGAAAG
ABCA1: AGTGATAATCAAAGTCAAAGGCACAC/AGCAACTTGGCACTAGTAACTCTG
ABCG1: TTTCCCAGAGATCCCTTTCA/ATCGAATTCAAGGACCTTTCC
ApoE: ACAGATCAGCTCGAGTGGCAAA/ATCTTGCGCAGGTGTGTGGAGA
CYP27A1: GGAGGGCAAGTACCCAATAAGA/TGCGATGAAGATCCCATAGGT
CYP27B1: TCAGCAGGCATCGCAGAAC/GCATTGGATCCTGAGGAATGA
